# Supplementary material for: Global identification of functional microRNA-mRNA interactions in Drosophila
Source: Nat Commun. 2019 Apr 9;10:1626. doi: 10.1038/s41467-019-09586-z (PMC6456604; doi:10.1038/s41467-019-09586-z)
Supplement: Supplementary file 1 — Supplementary Information [file 41467_2019_9586_MOESM1_ESM.pdf]

Global identification of functional microRNA-mRNA interactions in *Drosophila*

Wessels *et al*.

Supplementary Figures

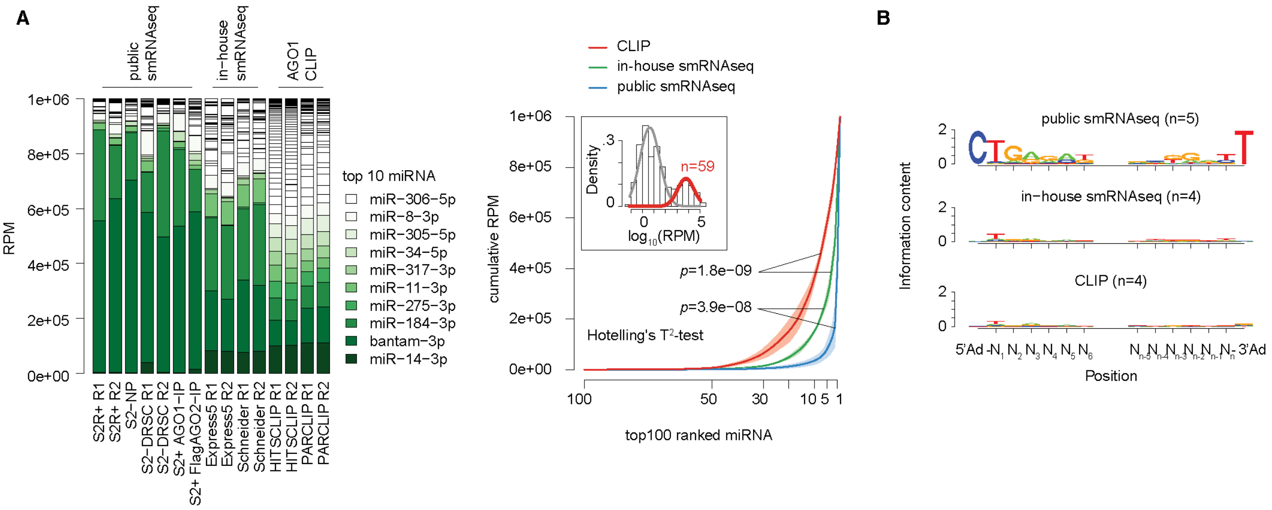

Supplementary Figure 1: A) As in Figure 1A. (Left) miRNA quantification in publicly available and in-house smRNA-seq samples (Supplementary Data 1). miRNA annotated reads were normalized to reads per million (RPM). Bar chart representing miRNA RPM across individual libraries and sorted by the mean RPM of miRNA quantified in CLIP libraries. (Right) Cumulative miRNA RPM distribution of top 100 detected and RPM-ranked miRNAs. The solid line represents the mean across libraries and shades represent standard deviation. The inset depicts $\log_{10}$ transformed CLIP-derived miRNA RPM that show a bimodal distribution. A mixture model was fitted identifying 59 ‘CLIP-enriched’ miRNA. B) Sequence Logo of read starts (nucleotide 1 to 6) and read ends (nucleotide n to n-5) preceded by the last nucleotide of 5’adapter (5’Ad) or followed by the first nucleotide of 3’adapter (3’Ad) used. All uniquely aligning reads were used. Public smRNA-seq libraries had non-variant adapter ends, while in-house smRNA-seq and CLIP libraries used randomized 4N nucleotide adapter ends.

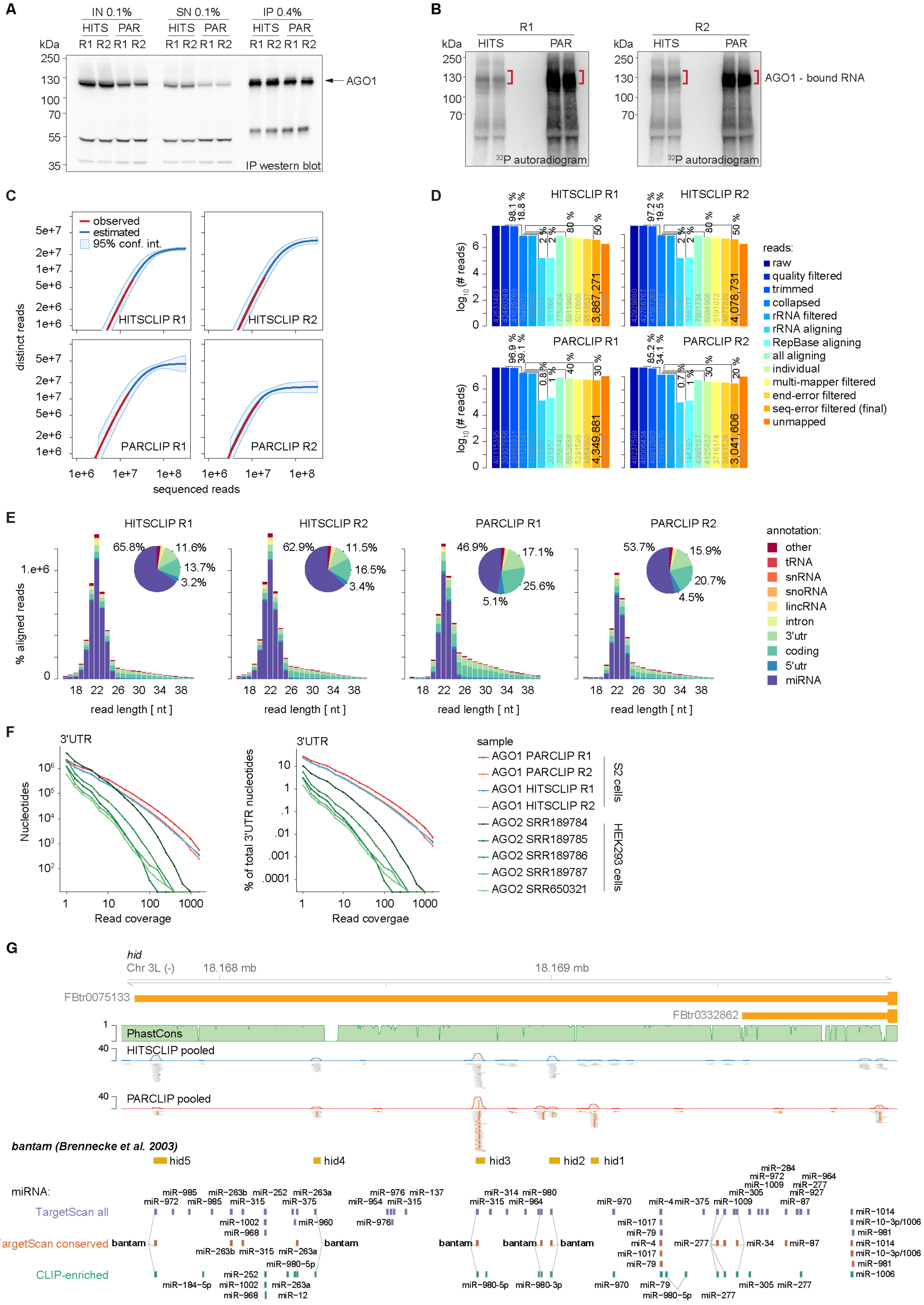

A
IN 0.1%
SN 0.1%
IP 0.4%
HITS PAR
R1 R2 R1 R2
kDa
250
130
100
70
55
35
AGO1
IP western blot
B
R1
R2
HITS PAR
AGO1 - bound RNA
32P autoradiogram
C
observed
estimated
95% conf. int.
HITSCLIP R1
HITSCLIP R2
PARCLIP R1
PARCLIP R2
distinct reads
sequenced reads
D
HITSCLIP R1
HITSCLIP R2
PARCLIP R1
PARCLIP R2
log10 (# reads)
3,867,271
4,078,731
4,349,881
3,041,606
reads:
raw
quality filtered
trimmed
collapsed
rRNA filtered
rRNA aligning
RepBase aligning
all aligning
individual
multi-mapper filtered
end-error filtered
seq-error filtered (final)
unmapped
E
HITSCLIP R1
HITSCLIP R2
PARCLIP R1
PARCLIP R2
% aligned reads
read length [nt]
65.8%
11.6%
13.7%
3.2%
62.9%
11.5%
16.5%
3.4%
46.9%
17.1%
25.6%
5.1%
53.7%
15.9%
20.7%
4.5%
annotation:
other
tRNA
snRNA
snoRNA
lincRNA
intron
3'utr
coding
5'utr
miRNA
F
3'UTR
Nucleotides
% of total 3'UTR nucleotides
Read coverage
Read covergae
sample
AGO1 PARCLIP R1
AGO1 PARCLIP R2
AGO1 HITSCLIP R1
AGO1 HITSCLIP R2
AGO2 SRR189784
AGO2 SRR189785
AGO2 SRR189786
AGO2 SRR189787
AGO2 SRR650321
S2 cells
HEK293 cells
G
hid
Chr 3L (-)
18.168 mb
18.169 mb
FBtr0075133
FBtr0332862
PhastCons
HITSCLIP pooled
PARCLIP pooled
bantam (Brennecke et al. 2003)
hid5
hid4
hid3
hid2
hid1
miRNA:
TargetScan all
TargetScan conserved
CLIP-enriched

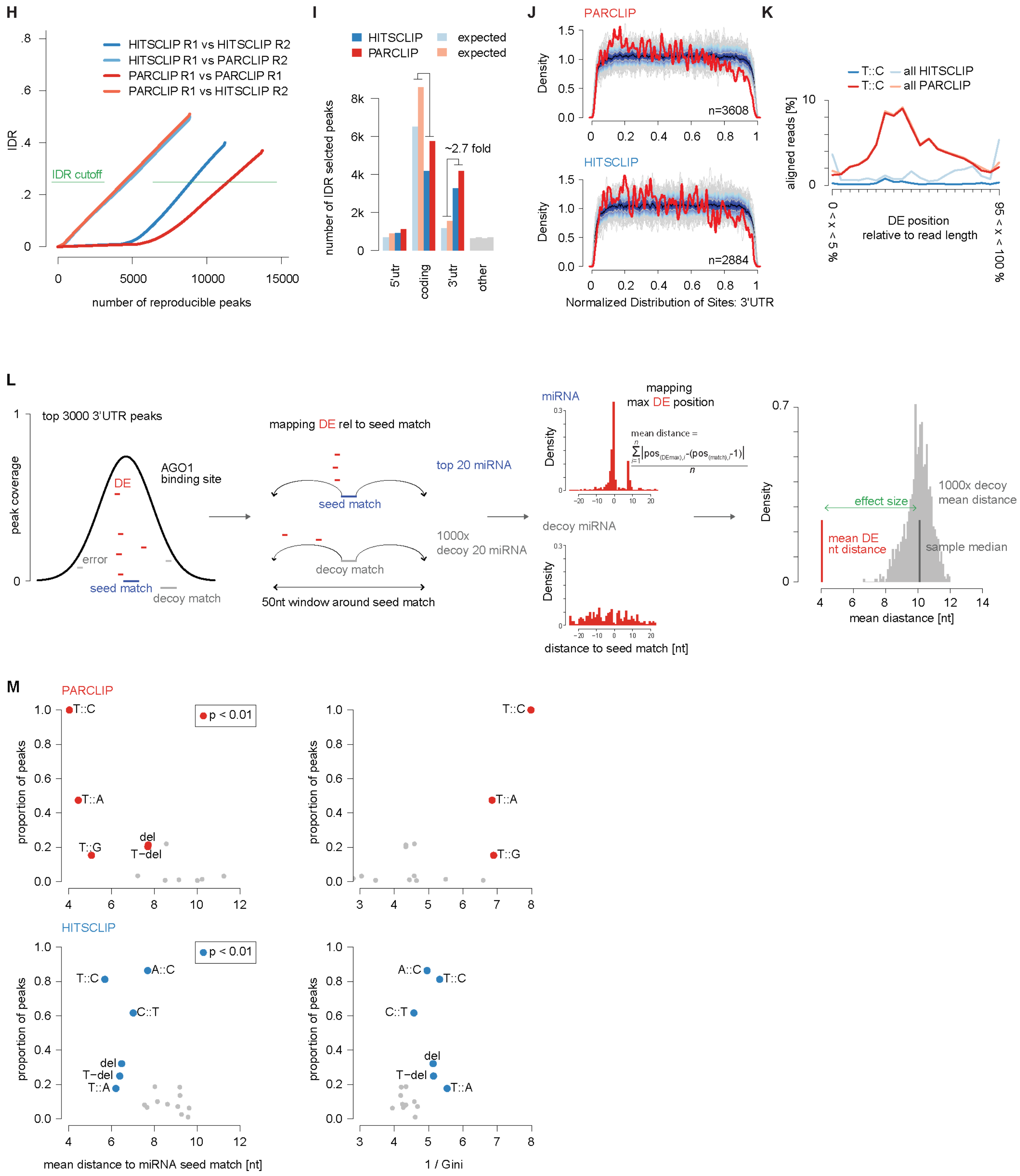

Supplementary Figure 2: A) IP western blot analysis for endogenous AGO1. IN = cleared lysate IP-input; SN = supernatant after immunoprecipitation; IP = aliquot of precipitate. Percentages indicate amounts of the total volume loaded. B) ^{32}P-autoradiogram of 5'-end-labelled RNP complexes separated after gel electrophoresis and transfer to a membrane. Red parentheses indicate AGO1-bound RNA fragments cloned during CLIP library generation. C) Library complexity analysis of AGO1 HITS- and PARCLIP libraries using Preseq [1]. D) Sample processing of AGO1 HITS- and

PARCLIP libraries. (raw = all sequenced reads; quality filtered = removed reads w/ uncalled bases and low quality; trimmed = after adapter trimming, min length incl. randomized adapter end = 24nt; collapsed = after duplicate removal; rRNA filtered = after filtering rRNA aligning reads; all aligning = total number of alignments; individual = unique number of aligned reads; multi-mapper filtered = after multi-mapper removal (used for microRNA quantification); end-error filtered = filtered reads with mis-alignments within the first or last 2 nt ; seq-error filtered = after removal of reads with mis-matches to the reference identified by evaluating read barcodes, genomic alignment coordinates and read copy number (used for comparative CLIP analysis)). E) Read annotation of relevant annotation categories. Pie charts depict the proportions of all reads. Bar chart depicts the total numbers of reads for a given read length, colored by annotation category. Here, we used multi-mapper filtered reads, as the final filtered alignments do not contain a substantial number of miRNA annotated reads with common untemplated 3' ends. F) 3'UTR read coverage of S2 cell HITSCLIP and PARCLIP samples, as well as five AGO2 PARCLIP libraries generated from HEK293 cells [2,3]. Y-axis represents the number of nucleotides (left) or the percentage of all 3'UTR nucleotides (right) that show a certain minimal read coverage (=number of reads uniquely mapped reads; x-axis). G) Browser shot of 3'UTR regions of the first described miRNA target in *D. melanogaster* for the gene *hid* (Brennecke et al. 2003). Depiction of AGO1 HITSCLIP (blue) and PARCLIP (red) coverage tracks (y-axis shows number of detected CLIP reads) including alignments along 3'UTRs as well as UCSC 27-way PhastCons scores (green). Red squares in individual read alignments indicate T-to-C mismatches to the dm6 reference. Red bars within coverage tracks indicate T-to-C conversion proportion at nucleotide resolution. Below, 7mer and 8mer seed matches for all detected miRNA (TargetScan 6.2 - conserved and non-conserved family info), conserved miRNA (TargetScan 6.2 - predicted conserved targets) and CLIP-enriched (see Supplementary Figure 1C) miRNA are indicated. Gold bars indicate original miRNA target site predictions. Endogenous AGO1-binding signal overlaps all five (hid1 - hid5) originally predicted bantam-3p matches. Only hid2 to hid5 contain miRNA seed match prediction for 7mer-1A, 7mer-m8 or 8mer-1A. Hid1 does not contain a prediction for bantam, because of a GU-wobble at seed match position 7. One originally not predicted bantam seed match between hid2 and hid3 shows AGO1 binding evidence for PARCLIP only. In addition, the most 5' AGO1 binding site contains a 6mer3-8 prediction, not indicated here. While seven bantam matches show binding evidence, other predicted miRNA binding sites lack this evidence. H) Irreproducible discovery rate of AGO1 HITSCLIP and PARCLIP Piranha peak calls. The green line indicates the IDR-cutoff chosen for downstream comparative analysis. I) Annotation of IDR-selected peaks relative to the numbers expected by chance given the median mRNA annotation feature length of expressed genes in S2 cells. J) Relative distribution of IDR-selected AGO1 binding sites in targeted 3'UTRs. K) Relative position of DEs (T-to-C conversions or all conversions) within all uniquely aligned sequencing reads in AGO1 HITSCLIP and PARCLIP. L) Scheme for testing diagnostic potential of individual nucleotide conversions or deletions for positional preference 5' proximal to unambiguous miRNA 7mer/8mer seed matches (see methods). M) Results according to Figure 2D and 2L). Scatterplot of mean distance to miRNA start (x-axis) relative to proportions of IDR-selected peaks (left). Alternatively, 1/Gini coefficient was calculated to indicate positional preferences at any distance relative to unambiguous miRNA seed matches (right).

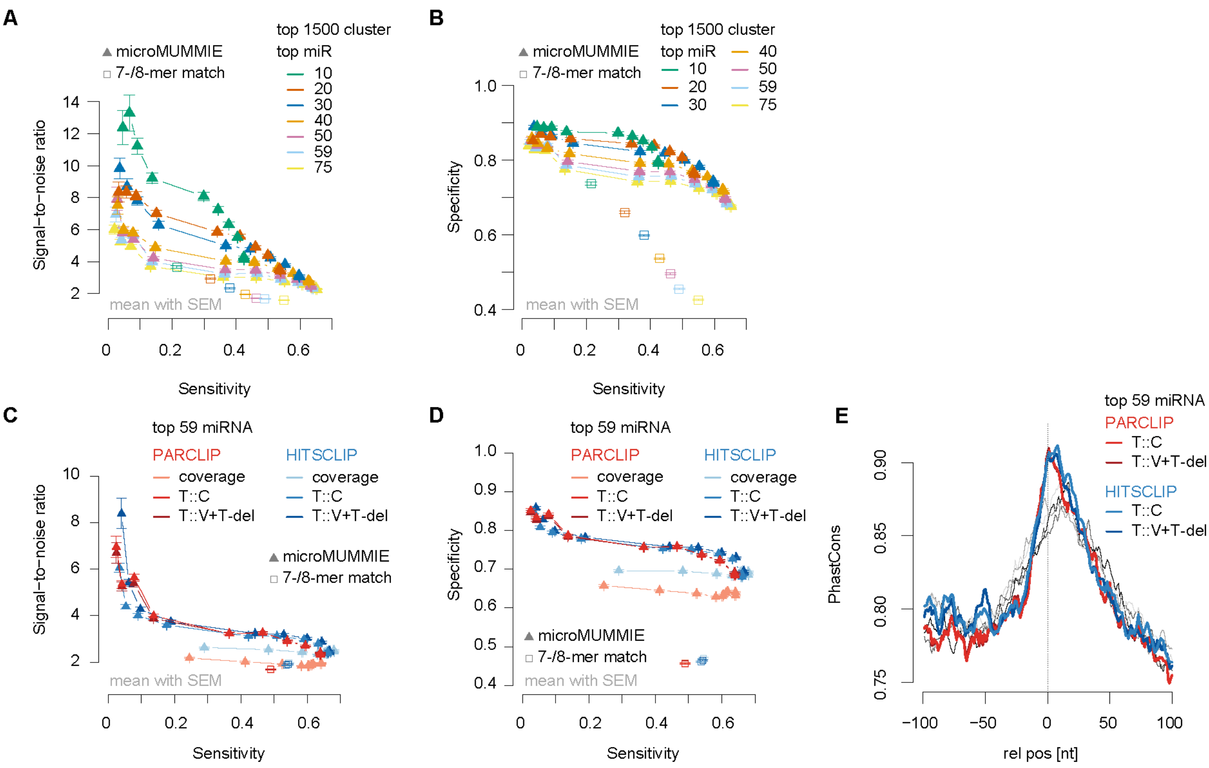

Supplementary Figure 3: A) Signal-to-noise estimate vs. sensitivity on the top 1500 AGO1 3'UTR clusters derived from AGO1 PARCLIP IDR-selected peaks. Here, miRNA seed matches were predicted for the top *n* CLIP-detected miRNA relative to the same number of shuffled decoy miRNA. The results are depicted as mean across 100 individual shuffling experiments, with error bars representing SEM. Individual triangles indicate changes in microMUMMIE variance levels. Squares show basic 7mer-A1, 7mer-m8 or 8mer-A1 matches anywhere within clusters. B) As in A), but depicting specificity estimates vs. sensitivity. C) As before, SNR estimate for HISTCLIP and PARCLIP derived DE signal for miRNA seed match prediction given the top 59 'CLIP-enriched' miRNA relative to 59 shuffled decoy miRNAs. X-axis depicts sensitivity. Coverage = inferred single nucleotide peak summit position. D) Similar to C) but depicting specificity vs. sensitivity. E) UCSC 27way PhastCons scores relative to the inferred crosslinked nucleotides for clusters with miRNA seed match (at microMUMMIE variance 0.01; Viterbi mode) prediction or a random nucleotide within the same peaks.

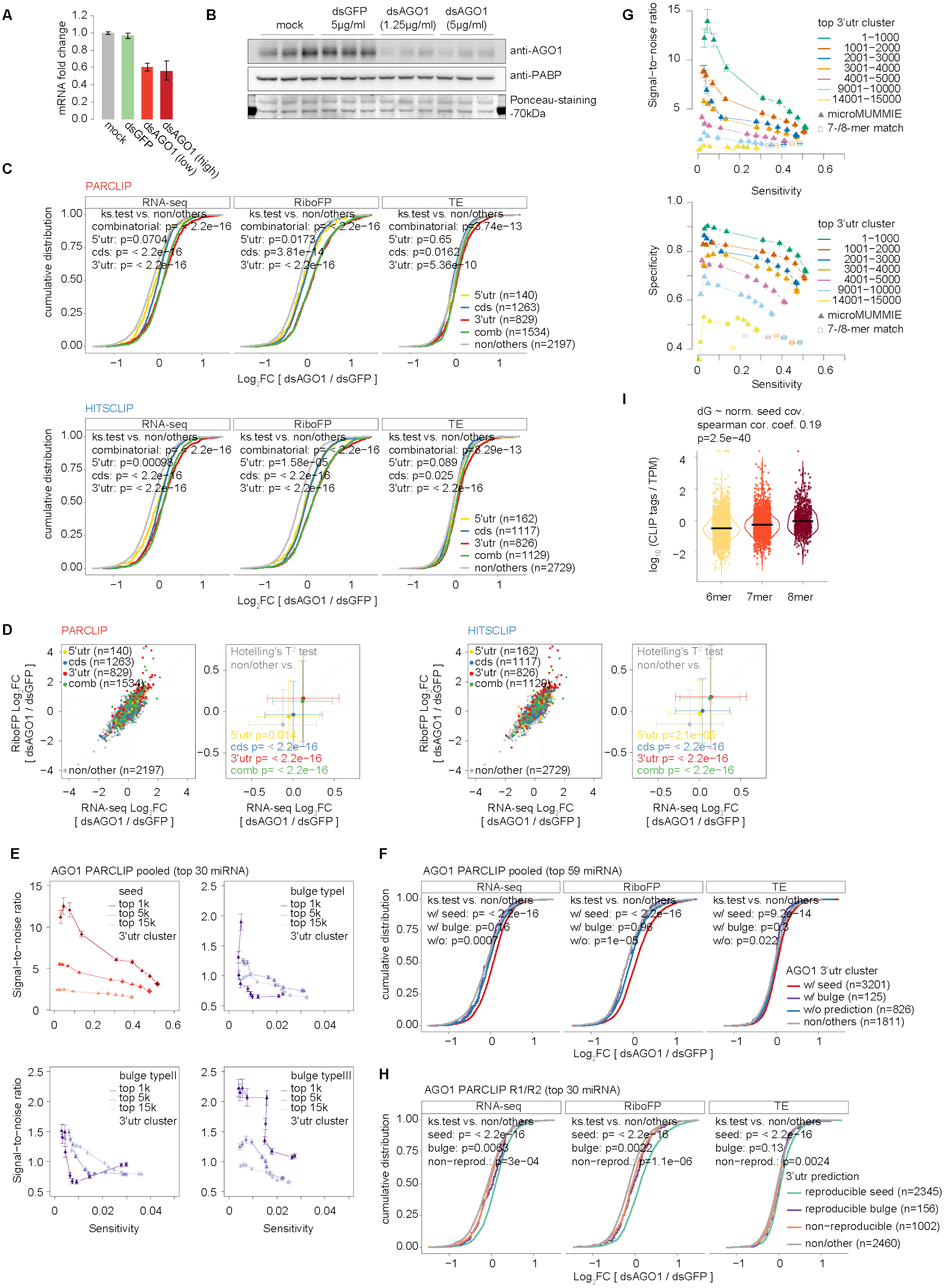
A
mRNA fold change
mock
dsGFP
dsAGO1 (low)
dsAGO1 (high)
B
mock
dsGFP 5µg/ml
dsAGO1 (1.25µg/ml)
dsAGO1 (5µg/ml)
anti-AGO1
anti-PABP
Ponceau-staining -70kDa
C
PARCLIP
RNA-seq
RiboFP
TE
ks.test vs. non/others
combinatorial: p= < 2.2e−16
5'utr: p=0.0704
cds: p= < 2.2e−16
3'utr: p= < 2.2e−16
combinatorial: p= < 2.2e−16
5'utr: p=0.0173
cds: p=3.81e−14
3'utr: p= < 2.2e−16
combinatorial: p=3.74e−13
5'utr: p=0.65
cds: p=0.0162
3'utr: p=5.36e−10
5'utr (n=140)
cds (n=1263)
3'utr (n=829)
comb (n=1534)
non/others (n=2197)
cumulative distribution
Log2FC [dsAGO1 / dsGFP]
HITSCLIP
combinatorial: p= < 2.2e−16
5'utr: p=0.00098
cds: p= < 2.2e−16
3'utr: p= < 2.2e−16
combinatorial: p= < 2.2e−16
5'utr: p=1.58e−05
cds: p= < 2.2e−16
3'utr: p= < 2.2e−16
combinatorial: p=8.29e−13
5'utr: p=0.089
cds: p=0.025
3'utr: p= < 2.2e−16
5'utr (n=162)
cds (n=1117)
3'utr (n=826)
comb (n=1129)
non/others (n=2729)
D
PARCLIP
5'utr (n=140)
cds (n=1263)
3'utr (n=829)
comb (n=1534)
non/other (n=2197)
Hotelling's T2 test
non/other vs.
5'utr p=0.014
cds p= < 2.2e−16
3'utr p= < 2.2e−16
comb p= < 2.2e−16
RiboFP Log2FC [dsAGO1 / dsGFP]
RNA-seq Log2FC [dsAGO1 / dsGFP]
HITSCLIP
5'utr (n=162)
cds (n=1117)
3'utr (n=826)
comb (n=1129)
non/other (n=2729)
5'utr p=2.1e−05
cds p= < 2.2e−16
3'utr p= < 2.2e−16
comb p= < 2.2e−16
E
AGO1 PARCLIP pooled (top 30 miRNA)
seed
bulge typeI
bulge typeII
bulge typeIII
top 1k
top 5k
top 15k
3'utr cluster
Signal−to−noise ratio
Sensitivity
F
AGO1 PARCLIP pooled (top 59 miRNA)
RNA-seq
RiboFP
TE
ks.test vs. non/others
w/ seed: p= < 2.2e−16
w/ bulge: p=0.16
w/o: p=0.0007
w/ seed: p= < 2.2e−16
w/ bulge: p=0.96
w/o: p=1e−05
w/ seed: p=9.2e−14
w/ bulge: p=0.3
w/o: p=0.022
AGO1 3'utr cluster
w/ seed (n=3201)
w/ bulge (n=125)
w/o prediction (n=826)
non/others (n=1811)
cumulative distribution
Log2FC [dsAGO1 / dsGFP]
G
Signal−to−noise ratio
Specificity
Sensitivity
top 3'utr cluster
1−1000
1001−2000
2001−3000
3001−4000
4001−5000
9001−10000
14001−15000
microMUMMIE
7-/8-mer match
H
AGO1 PARCLIP R1/R2 (top 30 miRNA)
RNA-seq
RiboFP
TE
ks.test vs. non/others
seed: p= < 2.2e−16
bulge: p=0.0063
non−reprod.: p=3e−04
seed: p= < 2.2e−16
bulge: p=0.0022
non−reprod.: p=1.1e−06
seed: p= < 2.2e−16
bulge: p=0.13
non−reprod.: p=0.0024
3'utr prediction
reproducible seed (n=2345)
reproducible bulge (n=156)
non−reproducible (n=1002)
non/other (n=2460)
cumulative distribution
Log2FC [dsAGO1 / dsGFP]
I
dG ~ norm. seed cov.
spearman cor. coef. 0.19
p=2.5e−40
log10 (CLIP tags / TPM)
6mer
7mer
8mer

Supplementary Figure 4: A) Real-time PCR AGO1 fold change relative to b-Tub84B for dsRNA knockdown of AGO1 (low = 1.25µg/ml; high = 5µg/ml) versus untreated (mock) or dsGFP (5µg/ml) treated S2 cells (n=3). B) Western blot analysis confirming AGO1 protein knockdown. Source data are provided as a Source Data file. Samples similar to A). C) Cumulative distribution showing RNA-seq, RiboFP and TE $\log_2$ fold changes of dsAGO1 (1.25µg/ml) vs. dsGFP (5µg/ml) treated samples for genes with IDR-selected peaks in PARCLIP and HITSCLIP. *n* represents the number of genes with peaks in 5'UTR, CDS, 3'UTR or a combination of two or more mRNA annotation categories. P value was calculated in a two-sided Kolmogorov-Smirnov test versus genes without peak. D) Same data as in C, scatterplot showing $\log_2$ fold changes of individual genes in RNA-seq vs. RiboFP according to peak annotation category, or the median and standard deviation of the selected gene population to indicate population weights. E) SNR estimate on the top n-thousand AGO1 3'UTR PARalyzer clusters from pooled AGO1 PARCLIP samples. Here miRNA seed matches as well as nucleation bulge type I, type II and type III were predicted for the top 30 CLIP-detected miRNAs relative to the same number of shuffled decoy miRNA. For bulges, only non-redundant sequences were used and evaluated in orphan clusters only (cluster w/o true miRNA seed match). The results are depicted as mean across 100 individual shuffling experiments, with error bars representing SEM. Individual triangles indicate changes in microMUMMIE variance levels. Only non-redundant nucleation bulges in orphan clusters were considered (see methods). Y-axis = Signal-to-noise ratio, x-axis = sensitivity. F) Cumulative distribution showing RNA-seq, RiboFP and TE $\log_2$ fold changes for genes with 3'UTR annotating PARalyzer cluster in pooled AGO1 PARCLIP samples either with seed (=w/ seed), with nucleation bulge (w/ bulge) or without (=w/o) a miRNA binding site prediction given 59 'CLIP-enriched' miRNAs, relative to genes without a 3'UTR cluster. P value was calculated in a two-sided Kolmogorov-Smirnov test versus genes with no 3'UTR cluster. G) As in E, SNR estimate on the top n-thousand AGO1 3'UTR PARalyzer clusters from pooled AGO1 PARCLIP samples. Here miRNA seed matches were predicted for the top 30 CLIP-detected miRNAs relative to the same number of shuffled decoy miRNA. (top) Signal-to-noise ratio vs. sensitivity, (bottom) specificity vs. sensitivity. H) Similar to F but comparing reproducible (in both AGO1 PARCLIP replicates) miRNA seed match predictions, reproducible nucleation bulge predictions versus predictions made in only one AGO1 PARCLIP replicate (= non-reproducible) for 3'UTR PARalyzer cluster. I) Violin plot showing the $\log_{10}$-transform ratio of seed match spanning reads and target gene TPM stratified by 6mer, 7mer and 8mer seed matches (according to Figure 4C). The black bar indicates the median. The spearmen correlation coefficient was calculated for the normalized seed coverage versus hybridization energy (dG) for a given seed match prediction.

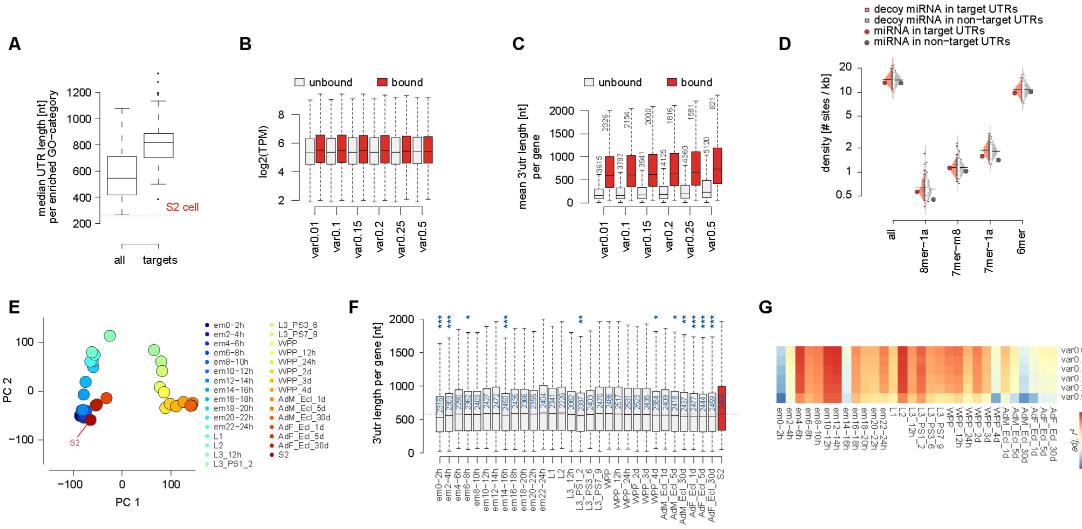

Supplementary Figure 5: A) Boxplot of median 3'UTR length of all genes and miRNA-targeted genes associated with enriched GOBP-terms from Supplementary Data 14. The red line represents the median 3'UTR length of all expressed gene in S2 cells. B) miRNA target gene expression levels and C) mean gene-wise 3'UTR lengths of miRNA target genes at different microMUMMIE stringency cutoffs. Transcript 3'UTR lengths were extracted from ensemble v81 GTF using the Bioconductor package Genomic Features [4]. Transcript isoform percentage was estimated using RSEM [5] for wildtype S2 cell total RNA-seq samples generated for this study. The mean gene 3'UTR length was calculated by multiplying transcript 3'UTR length times estimated isoform percentage. We considered only genes that have been reliably detected and have been used to calculate mRNA, ribosomal profiling and translational efficiency changes presented in (n=5963, in Supplementary Data 4). D) Putative miRNA motif density in expressed target (red) and non-targets (grey) 3'UTRs irrespective of AGO1-binding. Points represent the predicted miRNA motif density for the top30 CLIP-detected miRNAs. Split violins indicate the predicted miRNA motif density for dinucleotide-shuffled decoy-miRNA sequences. Black bars represent the mean decoy miRNA motif density of 100 individual shuffling experiments. miRNA motif density was normalized to the total target and non-target 3'UTR length. Here, the transcript isoform with the highest isoform percentage has been considered. E) Principle component analysis of DESeq2-normalized and rlog-transformed S2 cell and fly developmental stage gene expression data[6] using 1000 genes with the highest gene expression variance. F) 3'UTR length distributions of genes with reproducible miRNA target sites in S2 cells (TPM and read count > 5). Asterisks indicated significance between 3'UTR length of a given samples compared to 3'UTR length distributions observed for the target genes in S2 cells (two-sided Kolmogorov-Smirnoff test, Signif. codes: 0 '***' 0.001 '**' 0.01 '*' 0.05). G) Heatmap depicting the adj. R2 of gene-wise comparison of 3'UTR in embryonic samples compared to 3'UTR length in S2 samples for genes targeted by reproducible miRNA binding sites at different microMUMMIE stringency cutoffs. (Scale color code = adj. R2).

Supplementary Note 1

During smRNA-seq, RNA-seq and RiboFP read processing, we noted that a considerable number of unmapped reads aligned to viral genomes. Analyzing all smRNA-seq libraries for viral sequences, we found considerable populations of ~21 nucleotide (nt) long sequencing reads mapping to a recent collection of common *Drosophila* viruses [7] (Supplementary Note Figure 1A). These ~21nt long reads did not pile up in two closely spaced stacks reminiscent of processed miRNA, suggesting that these reads represent viral siRNAs. siRNAs are preferentially loaded into *Drosophila* AGO2 [8], and therefore were not present in AGO1 CLIP libraries (Supplementary Note 1B). Viruses within S2 cells could be a source of miRNAs or express genes that can be targeted by fly miRNAs and for example act a miRNA-sponge. In both cases, we would expect AGO1 HITSCLIP and PARCLIP reads to map as well to virus genomes. For each library, less than one percent of all collapsed CLIP reads mapped to the sum of all virus genomes (Supplementary Note Figure 1B). Reads that did map were specifically short (< 20 nt) and showed high alignment mismatch rates, suggesting that these alignments were no true viral sequences (Supplementary Note Figure 1B).

In order to assess if the virus presence will strongly affect the interpretation of our result, we compared our S2 cell RNA-seq samples to RNA-seq samples from 25 fly cell lines provided by the modENCODE consortium, plus additional S2 cell RNA-seq samples (Supplementary Data 16). More than ten other RNA-seq samples within these 47 available libraries also contained considerable proportions of reads mapping to viral genomes (Supplementary Note Figure 1C). We calculated gene expression counts for genes expressed in fly using HTSeq [9], and performed principal component analysis (PCA) on DESeq2 rlog-normalized gene expression counts for all genes that have conserved miRNA target sites identified by TargetScanFly [10]. PCA for genes targeted by the top 10 AGO1 CLIP-detected miRNA grouped in-house S2 cell RNA-seq samples together with all other S2 cell derived RNA-seq samples, suggesting high similarity between gene expression values of the selected miRNA target genes (Supplementary Note Figure 1D).

Thus, we can assume that gene expression changes observed in response to AGO1 depletion will be largely unaffected from present viruses.

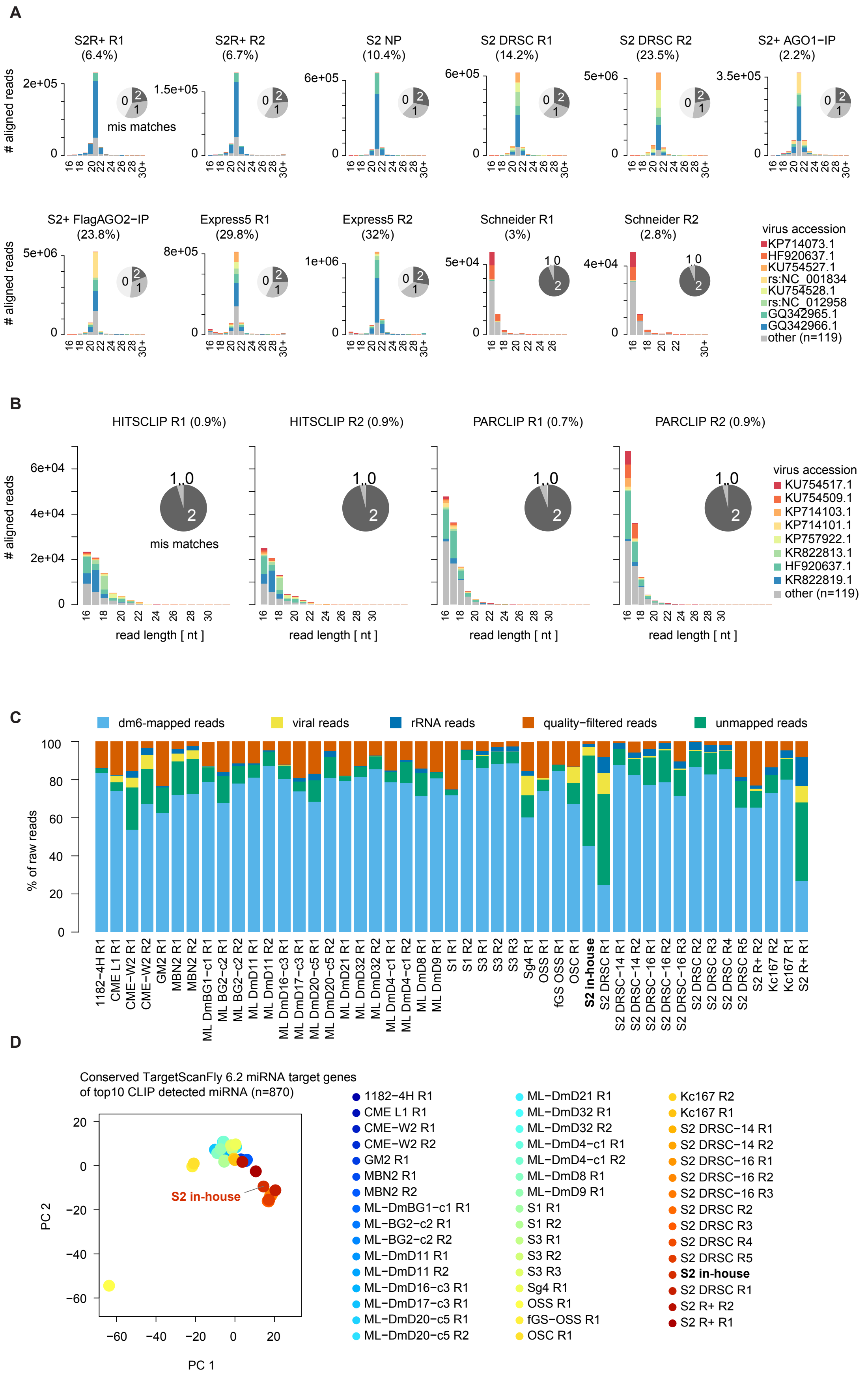

Supplementary Note Figure 1: A) and B) Read annotation overview of smRNA-seq (Supplementary Data 16) and AGO1 CLIP libraries mapping to *Drosophila* viruses [7], stratified

by read length. The 8 viruses with the most aligning reads across all CLIP samples are coloured, while the remaining 119 viruses are summarized in grey. Indicated percentages are relative to the total number of trimmed reads after PCR-duplicate removal (no PCR-duplicate removal for public smRNA-seq samples in A). The pie chart indicates the proportions of reads with a given number of mismatches to the reference virus sequences. C) Sample processing summary of available paired-end total and poly(A)-RNA-seq libraries, covering *Drosophila* cell lines processed by modENCODE [11] and additional samples from S2 cells (Supplementary Data 1). D) Principal component analysis of rlog-normalized gene expression counts. Selected are all 870 genes, that have conserved miRNA target sites for the top 10 AGO1-CLIP enriched miRNAs, as reported by TargetScanFly v6.2 [10].

Supplementary Methods

Biochemical methods

S2 cell handling

Drosophila Schneider2 (S2) cells were a generous gift from the Robert Zinzen lab (Max-Delbrueck-Center for Molecular Medicine). S2 cells were grown at 25 °C in ExpressFive SFM medium (*Life Technologies #10486025*) with 10 % heat-inactivated fetal bovine serum (FBS) (*Life Technologies #16000044*), 12 % L-Glutamine (*Life Technologies #25030024*) and 1 % Penicillin-Streptomycin (*Life Technologies #15070063*). All experiments have been conducted with the S2 cell sub-clone and culturing conditions described above. For small RNA sequencing we additionally sequenced S2 cells (*Life Technologies*, *#R69007*), cultured in Schneider's Cell medium w/ L-Glutamine (*SIGMA #S0146*).

Western blot

Western blot analyses were carried out under standard conditions. Samples were denatured with LDS sample buffer (*Life technologies #B0007*) including reducing agents and separated on 4-12% Bis-Tris polyacrylamide gels. For CLIP loading controls, RNP complexes were transferred to nitrocellulose membranes via semi-dry transfer using NuPAGE transfer buffer (*Life technologies #NP00061*) supplemented with 20% Methanol. For all other experiments, proteins were transferred with dry transfer using iBlot Gel Transfer Stacks Nitrocellulose (*Life technologies #IB301001*) following manufacturer's descriptions. Membranes were blocked with 2.5% Milk powder in 1xTBST buffer (0.1% Tween). Endogenous AGO1 was visualized using anti-AGO1 Abcam, ab5070). Probing with anti-PABP (generous gift from Marina Chekulaeva lab) and Ponceau staining of the membrane served as loading control.

Quantitative real-time PCR

For total RNA isolation cells were washed once in ice-cold PBS and lysed in 375µl TRIZOL (*Life Technologies #15596018*). Total RNA was isolated using Direct-zol RNA MiniPrep Kit (*Zymo #R2052*) following the manufacturer's description including on-column DNase digestion. Total RNA was reverse transcribed using the cDNA Synthesis Kit iScript (*BioRad #1708891*). Transcript knockdown was confirmed using Sso Fast Eva Green Supermix (*BioRad #1725202*) (Primer: Supplementary Data 15).

MiRNA Northern Blot analysis

Total RNA was isolated from $9*10^7$ S2 cells in 4.5 ml TRIZOL (*Life Technologies #15596018*) following manufacturer's descriptions. RNA concentrations were determined using Qubit RNA BR Assay (*Life Technologies #Q10211*). We loaded 30 µg total RNA next to a miRNA standard (Oligos: Supplementary Data 15) on a 1.5 mm 15 % TBE-UREA gel. The RNA was transferred to Amersham Hybond-N+ (*GE Healthcare #RPN303B*) membranes at <= 200mA and <= 20V for 6 hrs to overnight, and UV-crosslinked using a SpectroLinker XL1000 (twice 120 mJ/cm^2). After blocking the membrane with sonicated salmon sperm DNA (*AppliChem #A2159*), membranes were incubated with ^{32}P-labelled reverse-complement DNA oligos (Supplementary Data 15) overnight at Tm minus 10°C. After washing, the membranes were exposed for 48h. The radioactive signal was quantified using FIJI [12]. miRNA molecule numbers in the total RNA samples were estimated according to the linear regression standard model and divided by the total RNA amount per cell (6.02 pg/cell; sd = 1.28 pg/cell). The total RNA amount per cell was estimated measuring the total RNA yield from $0.5*10^6$, $1*10^6$ and $2*10^6$ cells purified with the Direct-zol RNA MiniPrep Kit (*Zymo #R2052*) following the manufacturer's description including on-column DNase digestion in triplicates.

SmallRNA sample preparation

SmallRNA libraries from samples used in miRNA northern blot experiments were generated using NEXTflex Small RNA Library Prep kit v3 (*Bioo Scientific #5132*), starting with 2 µg of total RNA and SRQC/ERDN-spike-in mix [13] (Supplementary Data 15; a generous gift from Dr. Timo Breit's lab at University of Amsterdam) using the following modifications: Following upon excessive 3' adapter removal the samples were eluted in 12 µl H_2O. 10.5 µl were transferred to a new tube and supplemented with 1 µl 2S rRNA antisense DNA-oligo (100 µM) (Oligo: Supplementary Data 15). 2S rRNA antisense DNA oligonucleotides were annealed (5 min 75 °C; 15 min 37 °C; 15 min 25 °C) to block short and abundant 2S rRNA during 5'adapter ligation [14]. 2S rRNA reads were decreased by up to 200-fold (without bloc ~65 % raw reads; with bloc ~0.4 % raw reads). Using pilot-PCR the minimal cycle number for the preparative smallRNA library was determined to 16-18 cycles. The amplicons were gel-purified and size-selected prior to sequencing on NextSeq500.

Gene knockdown

dsRNA-mediated knockdown was carried out as described before [15,16]. Specifically, *in vitro* transcription DNA templates were amplified from plasmid DNA using primers including a T7 site (Oligos: Supplementary Data 15). For *in vitro* transcription the MegaScript T7 kit (*Life technologies # AM1334*) was used following the manufacturer's description. S2 cells were treated with dsRNA (dsGFP 5µg/ml (in 2ml), dsAGO1 1.25µg/ml (low) or 5µg/ml (high)) or without (mock) for 72h. We included a lower dsRNA concentration for AGO1, as it yielded similar knockdown efficiency after 72 hours as compared to commonly used higher dsRNA amounts. Experiments were carried out in biological triplicates on different days, while collecting matched samples for total RNA, ribosomal footprinting and protein from a single replicate in parallel.

Ribosomal footprinting sample preparation

Ribosomal footprinting was carried out as described in [17], with minor changes. In brief, cells were rinsed from the plate and transferred to a 50 ml falcon tube, preloaded with Cycloheximide (*SIGMA #C4859*) for a final concentration of 0.1 mg/ml. Cells were spun down at 200g for 5 min at 4°C and washed once with cold PBS supplemented with 0.1 mg/ml CHX. After aspiration, the cell pellet was snap-frozen in liquid nitrogen. The subsequent lysis, footprinting and recovery of ribosome-protected fragments were carried out as described in [17]. Ribosomal footprinting libraries were then generated using the NEXTflex Small RNA Library Prep Kit (*Bioo Scientific #5132*) (CG6422 sample using Kit version 2, AGO1 sample using Kit version 3) using rRNA-depleted (RiboZero; *Illumina #MRZG12324*) and T4 PNK (*NEB #M0201*) treated RNA as input. By pilot-PCR the minimal PCR cycle number for library amplification was determined (CG6422 samples 17-20 cycles; AGO1 samples 14-15 cycles). Amplicons were gel-purified prior to sequencing a multiplexed pool on one flow cell (CG6422: HiSeq2000, 51 cycles single-end AGO1: NextSeq500 high output mode, 75 cycles single-end.)

RNA-seq sample preparation

To 2.5 µg total RNA input we added ERCC spike-ins [18] (AGO1 knockdown Experiment: mock and dsGFP: ERCC Mix-1; dsAGO1 low and high: ERCC Mix-2 (*Life technologies #4456740 #4456739*); 5µl of a 1:100 dilution), prior to rRNA depletion using RiboZero (*Illumina #MRZG12324*). 100ng rRNA-depleted RNA served as an

input for RNA-seq libraries generated using the NEXTflex Rapid Directional qRNA-Seq kit (*Bioo Scientific #5130*) following manufacturer's descriptions. AGO1 RNA-seq libraries were sequenced single-end with 75nt cycles as a multiplexed pool on NextSeq500.

Computational methods

Mapping reads to the genome

For genome mapping we used the dm6 genome build provided by ensembl (v81). Mapping tools and parameters were chosen with respect to data type and application. For all custom genome mapping (smallRNA-seq, HITS/PARCLIP, Ribosomal footprinting, single-end and paired-end RNA-seq) we used STAR v2.4.2a [19]. For PARCLIP data processing within PARpipe (https://github.com/ohlerlab/PARpipe) and estimation of library complexity and we used bowtie v1.1.2 [20]

Genome alignment for short reads:
Bowtie: -v 1 -m 10 --best –strata
STAR: --alignEndsType EndToEnd --runThreadN 4 --outFilterMultimapNmax 10 --outSAMattributes All --outFilterIntronMotifs RemoveNoncanonical --outReadsUnmapped Fastx --alignSJoverhangMin 12 --outFilterMatchNmin 15 --outFilterMismatchNmax 1 --outFilterMismatchNoverLmax 0.05 --outFilterMultimapScoreRange 3 --alignIntronMax 20000 --seedMultimapNmax 200000 --seedPerReadNmax 30000

Genome alignment for long reads:
--chimSegmentMin 30 --chimJunctionOverhangMin 30 --outFilterMultimapNmax 20 --outFilterMismatchNmax 999 --outFilterMismatchNoverLmax 0.04 --outFilterType BySJout --alignIntronMin 20 --outSAMattributes All --outFilterMatchNmin 20 --alignIntronMax 100000 --alignMatesGapMax 1000000 --alignSJDBoverhangMin 3 --outFilterIntronMotifs RemoveNoncanonicalUnannotated --alignSJoverhangMin 20

For human AGO2 PARCLIP libraries analyzed with PARpipe, reads were aligned to GRCh37.p13 with Gencode v19 annotation, using the same bowtie parameters.

For filtering and quantifying reads mapping to rRNA sequences (ensembl v81), repeat elements (extracted from RepBase v21 [21]) or common *Drosophila* viruses [7], we used either bowtie v1.1.2 [20] or STAR v2.4.2a [19].

Bowtie: -p 4 -q (-X 1000) --fr –best
STAR: --alignEndsType EndToEnd --runThreadN 4 --outFilterMultimapNmax 100 --outSAMattributes All --outFilterIntronMotifs RemoveNoncanonical --

alignSJoverhangMin 12 --outFilterMatchNmin 15 --outFilterMismatchNmax 1 --outFilterMismatchNoverLmax 0.05 --outFilterMultimapScoreRange 3 --alignIntronMax 20000 --seedMultimapNmax 200000 --seedPerReadNmax 30000

CLIP library quality assessment

To assess AGO1 CLIP library complexity, reads were adapter-trimmed and trimmed of randomized nucleotides retaining only trimmed reads of minimally 20nt using cutadapt [--discard-untrimmed --overlap=3 -n 1 –m 20] [22]. Reads were aligned using bowtie. Preseq was used with default setting to calculate expected and extrapolate yields [1,23].

To assess relative 3'UTR coverage AGO CLIP libraries, we processed Human AGO2 PARCLIP data sets have been processed with PARpipe as described above. For both *Drosophila* dm6 ensemble v81 and human Gencode v19 all annotated 3'UTRs were selected using the Bioconductor GenomicFeatures package [4]. Coverage tracks spanning selected 3'UTRs were systematically sliced imitating a global coverage cut-off allowed for quantifying the number of covered 3'UTR nucleotides. Global normalization between human and fly AGO CLIP samples could not be applied, as there is no obvious genome dependent scaling factor. Instead, the total number 3'UTR nucleotides was used to illustrate the relative covered 3'UTR space. Furthermore, differences in relative coverage as a result of sequencing depth cannot be excluded here.

Read, peak and cluster annotation

Annotation was done as described previously [24,25]. Briefly, for annotation we used annotate.pl, and annotation rank file from PARpipe (https://github.com/ohlerlab/PARpipe) and assigned annotation labels based on information provided by the dm6 ensemble v81 GTF. Repeat element information was extracted from UCSC and considered during annotation. For human AGO2 PARCLIP data sets, Gencode v19 annotation was applied.

For miRNAs reads were annotated to miRNA genes as described above first and intersected with mature miRNA coordinates retrieved from miRBase v21 after coordinate liftover from dm3 to dm6 [26].

Spatial and numerical peak enrichments

We ran RSEM v1.2.31 [5] using default settings accounting for strandedness on wild type stranded single strand RNA-seq samples from mock treated S2 cells and selected genes with a TPM > 1 present in fly dm6 ensembl v81. For each gene, we selected the transcript isoform with the highest isoform percentage or chose one randomly in case of ties. The list of selected transcript isoforms was used to calculate the median 5'UTR, CDS and 3'UTR length proportions using R Bioconductor packages GenomicFeatures and GenomicRanges [4]. Enrichments were calculated relative to median feature proportions (5'UTR=0.08 (131nt), CDS=0.78 (1309.5nt), 3'UTR=0.14 (234nt)).

Spatial preferences within 3'UTRs were determined using Spatial.pl and Spatial.R as part of PARpipe (https://github.com/ohlerlab/PARpipe) for IDR-selected peaks.

RNA-seq data processing

RNA sequencing reads of newly generated samples were 3' end quality-filtered and quality-trimmed using the fastx toolkit (http://hannonlab.cshl.edu/fastx_toolkit/) [quality filter: -q 25 -p 30; quality trimmer: -t 25 -l 30], as well as 5'end trimmed [trimmer: -f 13]. rRNA and common viral sequences were filtered out. The remaining reads were aligned to the dm6 genome using ensembl v81 annotation. We used umi-tools [27] to make optimal use of the UMIs present in the RNA sequencing data and the ERCC dashboard R Bioconductor package [28] to recover expected fold changes from the ERCC spike-in mixes in AGO1 libraries. As we could recover expected fold changes from the ERCC spike-in mixes more robustly without using the sequencing read UMIs, we disregarded the UMIs throughout our analysis and did not collapse the RNA-seq data, but instead trimmed the first 13 nucleotides (the first base of balanced nucleotide composition) of each read as indicated above.

Ribosomal footprinting data processing

Ribosome profiling sequencing reads were trimmed using cutadapt [22] [-m 18; --discard-untrimmed]. After collapsing the sequencing reads using the fastx toolkit (http://hannonlab.cshl.edu/fastx_toolkit/), we removed the four randomized nucleotides from both ends of the reads. After removal of rRNA sequences, the remaining reads were aligned to the fly genome. We confirmed expected periodicity of ribosome protected fragments using RiboTaper [17].

Differential Expression and Translational efficiency analysis

Raw counts on the ensembl v81 dm6 gene model were calculated for RNA-seq using HTSeq [-m union] [9] across mature transcripts, while ribosomal footprinting counts were generated on coding regions only using R Bioconductor packages (GenomicFeatures, GenomicAlignments [summarizeOverlaps with mode="Union"]) [4]. Xtail [29] was used to return $\log_2$ fold changes for translational efficiency, RNA expression levels and changes in ribosomal footprinting [bins = 10000, ci = .95]. For AGO1 experiments, only genes with > 1 count per million sequenced reads in all treatments and replicates in RNA-seq data as well as in ribosomal footprinting data were considered. The data shown represents the contrast between low dsAGO1 treatment at 1.25 µg/ml and dsGFP control treatment.

Assessing cell type similarity

To assess the similarity between in-house S2 cells and other fly cell lines based on gene expression profiles we retrieved paired-end RNA-seq samples from S2 cells and other fly cell lines from the short-read archive (SRA), and processed all in parallel as described above, with minor differences (Supplementary Data 16). In brief, RNA-seq libraries differed read lengths (>100 nt; 100nt; 75nt; 36nt). For long read samples, we trimmed all reads to remain at 66nt length. For >75 nt, read length was trimmed from 3'end and 5'end, samples of 75 nt length were trimmed from its 5'end only, while short read samples were not trimmed. Subsequently, reads were quality-trimmed from their 3'end using cutadapt [-q 20] [22], requiring a minimal read length of 30nt (20nt for 36nt RNA-seq samples). rRNA and common viral sequences were filtered out. The remaining reads were aligned to the dm6 genome. Gene expression counts were quantified using HTSeq [-union] [9], using the ensembl v81 dm6 gene model. Read counts were rlog-transformed using DESeq2 [30]. Genomic locations from conserved miRNA targets (TargetScanFly v6.2 [31]) were extracted via UCSC from TargetScanFly (i.e. http://targetscan.org/fly_12/ucsc/dm3/dm3ConsChr2L.bed) and lifted over to dm6 (https://genome.ucsc.edu/cgi-bin/hgLiftOver). Genes overlapping conserved miRNA target sites of the top 10 expressed miRNA were used as input for principal component analysis (PCA).

Information about transcriptional cell type similarity together with cellular virus burden can be found in Supplementary Note 1.

Assessing 3'UTR length and 3'UTR miRNA motif density

Transcript 3'UTR lengths were extracted from ensemble v81 GTF using the Bioconductor package Genomic Features [4]. Transcript isoform percentage was estimated using RSEM [5] for wildtype S2 cell total RNA-seq samples generated for this study and for the modENCODE developmental time course RNA-seq gene expression data [6] (only samples generated in Graveley lab have been considered) as described above. The mean gene 3'UTR length per gene was calculated by multiplying transcript 3'UTR length times estimated isoform proportions for analysis presented in Supplementary Figures 5A-C and 5F-G. To estimate putative miRNA motif density (Supplementary Figure 5D), we selected the 3'UTR from the transcript isoform with the highest isoform percentage or chose one randomly in case of ties. We considered only genes that have been reliably detected and have been used to calculate mRNA, ribosomal profiling and translational efficiency changes presented in (n=5963, in Supplementary Data 4). When replicates sequencing data were available, we processed each sample separately and averaged 3'UTR length estimates per biological sample in the end.

Putative miRNA motifs have been called using available perl scripts from target-scan[32] searching for all motifs without considering conservation. We searched for miRNA binding sites in selected 3'UTRs (see above) for the top30 CLIP-detected miRNAs and 100 individual di-nucleotide shuffled decoy sequences (one decoy sequence per true miRNA sequence). miRNAs of the same miRNA family have been reduced to one seed. miRNA motif density was normalized to the total target and non-target 3'UTR length.

Normalizing miRNA seed coverage and hybridization energy

To calculate miRNA seed coverage, all AGO1 PARCLIP reads used by PARalyzer overlapping the miRNA target site prediction were counted using summarizeOverlaps [mode = "union"] function from the Bioconductor package GenomicAlignments [4]. The seed coverage was normalized by dividing the read count with the target gene's TPM gene expression value.

To calculate the hybridization energies between miRNA and target, we used a simplified nearest neighbor model [33] and focused on the seed regions and disregarded potential supplementary 3'end pairing. We calculated hybridization energies between nucleotide duplets sliding nucleotide by nucleotide along the seed (Neighbor pairs [kJ/mol]: AA = -4.26, TT=4.26, AT = -3.67, TA = -2.5, CA = -6.12, AC = -6.12, GT = -

6.09, TG = -6.09, TC = -5.4, CT = -5.4, GA = -5.51, AG = -5.51, CG = -9.07, GC = -9.36, GG = -7.66, CC = -7.66; Terminal bases [kJ/mol]: G = 4.05, C = 4.05, A = 4.31, T = 4.31).

Gene set enrichment analysis

Gene ontology analysis on miRNA target genes shown in figure 5, was performed using the R Bioconductor package topGO [34]. We tested miRNA targets (all miR: n=2,601), the top decile of genes upregulated on mRNA level (mRNA), ribosomal footprinting level (RiboFP) and translational efficiency level (TE) upon AGO1-depletion (n=597), and all individual miRNA target sets, relative to all genes considered during functional analysis previously (n=5,962). Enriched GO-terms for all miRNA targets ($p < 0.001$, fisher's exact test, n=501) were with the corresponding enrichment p-values of the individual sets, as we did not observe strongly enriched GO-terms for individual miRNA target sets, which were not already covered by enrichments in all miRNA targets. P-values were $-\log_{10}$ transformed and GO-term-wise clustered (distance = “euclidean”, clustering = “ward”), and visualized using aheatmap from the NMF R package [35]. To calculate pair-wise similarities between enriched GO-terms of individual miRNA targetomes, we used mgoSim (parameters: measure = "Wang", ont = "BP", combine = "BMA") from the R package GOSemSim [36] for the top100 enriched terms ($p < 0.001$, fisher's exact test). The similarity scores were clustered as before (distance = euclidean, clustering = ward).

Visualization of sequencing data

For visualization we used Gviz [37] on either library size normalized bigwig files indicating differences in coverage (Figure 1B) or alignment files to visualize single nucleotide diagnostic events (Figure 2A, B and Supplementary Figure 2G).

Supplementary References

1. Daley, T. & Smith, A. D. Predicting the molecular complexity of sequencing libraries. *Nat. Methods* **10,** 325–7 (2013).
2. Kishore, S. *et al.* A quantitative analysis of CLIP methods for identifying binding sites of RNA-binding proteins. *Nat. Methods* **8,** 559–64 (2011).
3. Memczak, S. *et al.* Circular RNAs are a large class of animal RNAs with regulatory potency. *Nature* **495,** 333–338 (2013).
4. Lawrence, M. *et al.* Software for Computing and Annotating Genomic Ranges. *PLoS Comput. Biol.* **9,** 1–10 (2013).
5. Li, B. & Dewey, C. N. RSEM: accurate transcript quantification from RNA-Seq data with or without a reference genome. *Genome Biol.* **12,** 1–16 (2011).
6. Graveley, B. R. *et al.* The developmental transcriptome of Drosophila melanogaster. *Nature* **471,** 473–9 (2011).
7. Webster, C. L., Longdon, B., Lewis, S. H. & Obbard, D. Twenty-five new viruses associated with the drosophilidae. *Evol. Bioinforma.* **12,** 13–25 (2016).
8. Czech, B. *et al.* An endogenous small interfering RNA pathway in Drosophila. *Nature* **453,** 798–802 (2008).
9. Anders, S., Pyl, P. T. & Huber, W. HTSeq-A Python framework to work with high-throughput sequencing data. *Bioinformatics* **31,** 166–169 (2015).
10. Ruby, J. G. *et al.* Evolution, biogenesis, expression, and target predictions of a substantially expanded set of Drosophila microRNAs. *Genome Res.* **17,** 1850–64 (2007).
11. Cherbas, L. *et al.* The transcriptional diversity of 25 Drosophila cell lines. *Genome Res.* **21,** 301–314 (2011).
12. Schindelin, J. *et al.* Fiji: an open-source platform for biological-image analysis. *Nat. Methods* **9,** 676–682 (2012).
13. Locati, M. D. *et al.* Improving small RNA-seq by using a synthetic spike-in set for size-range quality control together with a set for data normalization. *Nucleic Acids Res.* **43,** (2015).
14. Wickersheim, M. L. & Blumenstiel, J. P. Terminator oligo blocking efficiently eliminates rRNA from Drosophila small RNA sequencing libraries. *Biotechniques* **55,** 269–272 (2013).
15. Clemens, J. C. *et al.* Use of double-stranded RNA interference in Drosophila cell lines to dissect signal transduction pathways. *Proc. Natl. Acad. Sci. U. S. A.* **97,** 6499–503 (2000).
16. Worby, C. a., Simonson-Leff, N. & Dixon, J. E. RNA Interference of Gene Expression (RNAi) in Cultured Drosophila Cells. *Sci. Signal.* **2001,** 1–8 (2001).
17. Calviello, L. *et al.* Detecting actively translated open reading frames in ribosome profiling data. *Nat. Methods* **13,** 165–169 (2016).
18. External RNA Controls Consortium. Proposed methods for testing and selecting the ERCC external RNA controls. *BMC Genomics* **6,** 150 (2005).
19. Dobin, A. *et al.* STAR: ultrafast universal RNA-seq aligner. *Bioinformatics* **29,** 15–21 (2013).
20. Langmead, B., Trapnell, C., Pop, M. & Salzberg, S. L. Ultrafast and memory-efficient alignment of short DNA sequences to the human genome. *Genome*

Biol. **10,** (2009).
21. Bao, W., Kojima, K. K. & Kohany, O. Repbase Update, a database of repetitive elements in eukaryotic genomes. *Mob. DNA* **6,** 11 (2015).
22. Martin, M. Cutadapt removes adapter sequences from high-throughput sequencing reads. *EMBnet.journal* **17,** 5–7 (2011).
23. Daley, T. & Smith, A. D. Modeling genome coverage in single-cell sequencing. *Bioinformatics* **30,** 3159–3165 (2014).
24. Mukherjee, N. *et al.* Global target mRNA specification and regulation by the RNA-binding protein ZFP36. *Genome Biol.* **15,** R12 (2014).
25. Ascano, M. *et al.* FMRP targets distinct mRNA sequence elements to regulate protein expression. *Nature* **492,** 382–386 (2012).
26. Kozomara, A. & Griffiths-Jones, S. MiRBase: Annotating high confidence microRNAs using deep sequencing data. *Nucleic Acids Res.* **42,** 68–73 (2014).
27. Smith, T., Heger, A. & Sudbery, I. UMI-tools : modeling sequencing errors in Unique Molecular Identifiers to improve quantification accuracy. *Genome Res.* **27,** 491–499 (2017).
28. Munro, S. a *et al.* Assessing technical performance in differential gene expression experiments with external spike-in RNA control ratio mixtures. *Nat. Commun.* **5,** 5125 (2014).
29. Xiao, Z., Zou, Q., Liu, Y. & Yang, X. Genome-wide assessment of differential translations with ribosome profiling data. *Nat. Commun.* **7,** 11194 (2016).
30. Love, M. I., Huber, W. & Anders, S. Moderated estimation of fold change and dispersion for RNA-seq data with DESeq2. *Genome Biol.* **15,** 550 (2014).
31. Kheradpour, P., Stark, A., Roy, S. & Kellis, M. Reliable prediction of regulator targets using 12 Drosophila genomes. *Genome Res.* **17,** 1919–1931 (2007).
32. Agarwal, V., Bell, G. W., Nam, J.-W. & Bartel, D. P. Predicting effective microRNA target sites in mammalian mRNAs. *Elife* **4,** 1–38 (2015).
33. SantaLucia, J. A unified view of polymer, dumbbell, and oligonucleotide DNA nearest-neighbor thermodynamics. *Proc. Natl. Acad. Sci.* **95,** 1460–1465 (1998).
34. Alexa, A. & Rahnenfuhrer, J. topGO: Enrichment analysis for Gene Ontology v2.22.0. (2010).
35. Gaujoux, R. & Seoighe, C. A flexible R package for nonnegative matrix factorization. *BMC Bioinformatics* **11,** 367 (2010).
36. Yu, G. *et al.* GOSemSim: An R package for measuring semantic similarity among GO terms and gene products. *Bioinformatics* **26,** 976–978 (2010).
37. Hahne, F. & Ivanek, R. Visualizing Genomic Data Using Gviz and Bioconductor. in *Statistical Genomics: Methods and Protocols* (eds. Math, E. & Davis, S.) 335–351 (Springer New York, 2016). doi:10.1007/978-1-4939-3578-9_16
