# Supplementary material for: Global identification of functional microRNA-mRNA interactions in Drosophila
Source: Nat Commun. 2019 Apr 9;10:1626. doi: 10.1038/s41467-019-09586-z (PMC6456604; doi:10.1038/s41467-019-09586-z)
Supplement: Supplementary file 3 — Description of Additional Supplementary Files [file 41467_2019_9586_MOESM3_ESM.pdf]

Description of Additional Supplementary Files

File Name: Supplementary Data 1
Description: miRNA quantification

File Name: Supplementary Data 2
Description: AGO1 HITSCLIP IDR-selected peaks

File Name: Supplementary Data 3
Description: AGO1 PARCLIP IDR-selected peaks

File Name: Supplementary Data 4
Description: Xtail results [dsRNA AGO1-low / dsRNA GFP]

File Name: Supplementary Data 5
Description: PARalyzer-Cluster on AGO1 PARCLIP R1 sample

File Name: Supplementary Data 6
Description: PARalyzer-Cluster on AGO1 PARCLIP R2 sample

File Name: Supplementary Data 7
Description: PARalyzer-Cluster on pooled AGO1 PARCLIP samples

File Name: Supplementary Data 8
Description: Comprehensive miRNA binding site set (3'UTR, top59 miRNA, on pooled AGO1 PAR-CLIP samples)

File Name: Supplementary Data 9
Description: Reproducible miRNA binding sites on separate AGO1 PAR-CLIP replicate samples (3'UTR, top59 miRNA)

File Name: Supplementary Data 10
Description: High confidence miRNA binding site set. Reproducible miRNA binding sites on separate AGO1 PAR-CLIP replicate samples (3'UTR, top30 miRNA)

File Name: Supplementary Data 11
Description: Reproducible miRNA nucleation bulge binding sites on separate AGO1 PAR-CLIP replicate samples (3'UTR, top30 miRNA)

File Name: Supplementary Data 12
Description: Reproducible miRNA binding sites on separate AGO1 PAR-CLIP replicate samples (coding regions, top30 miRNA)

File Name: Supplementary Data 13
Description: Reproducible miRNA binding sites on separate AGO1 PAR-CLIP replicate samples (5'UTR, top30 miRNA)

File Name: Supplementary Data 14
Description: Gene ontology enrichments

File Name: Supplementary Data 15
Description: Oligonucleotides and SQRC-ERDN spike-in information

File Name: Supplementary Data 16
Description: Process Data set statistics
