# Supplementary material for: Global identification of functional microRNA-mRNA interactions in Drosophila
Source: Nat Commun. 2019 Apr 9;10:1626. doi: 10.1038/s41467-019-09586-z (PMC6456604; doi:10.1038/s41467-019-09586-z)
Supplement: Supplementary file 20 — Reporting Summary [file 41467_2019_9586_MOESM20_ESM.pdf]

nature research

Corresponding author(s): Uwe Ohler

Last updated by author(s): Feb 13, 2019

# Reporting Summary

Nature Research wishes to improve the reproducibility of the work that we publish. This form provides structure for consistency and transparency in reporting. For further information on Nature Research policies, see Authors & Referees and the Editorial Policy Checklist.

## Statistics

For all statistical analyses, confirm that the following items are present in the figure legend, table legend, main text, or Methods section.

| n/a | Confirmed | |
|---|---|---|
| ☐ | ☒ | The exact sample size (*n*) for each experimental group/condition, given as a discrete number and unit of measurement |
| ☐ | ☒ | A statement on whether measurements were taken from distinct samples or whether the same sample was measured repeatedly |
| ☐ | ☒ | The statistical test(s) used AND whether they are one- or two-sided<br>*Only common tests should be described solely by name; describe more complex techniques in the Methods section.* |
| ☒ | ☐ | A description of all covariates tested |
| ☒ | ☐ | A description of any assumptions or corrections, such as tests of normality and adjustment for multiple comparisons |
| ☐ | ☒ | A full description of the statistical parameters including central tendency (e.g. means) or other basic estimates (e.g. regression coefficient) AND variation (e.g. standard deviation) or associated estimates of uncertainty (e.g. confidence intervals) |
| ☒ | ☐ | For null hypothesis testing, the test statistic (e.g. *F*, *t*, *r*) with confidence intervals, effect sizes, degrees of freedom and *P* value noted<br>*Give P values as exact values whenever suitable.* |
| ☒ | ☐ | For Bayesian analysis, information on the choice of priors and Markov chain Monte Carlo settings |
| ☒ | ☐ | For hierarchical and complex designs, identification of the appropriate level for tests and full reporting of outcomes |
| ☐ | ☒ | Estimates of effect sizes (e.g. Cohen's *d*, Pearson's *r*), indicating how they were calculated |

*Our web collection on statistics for biologists contains articles on many of the points above.*

## Software and code

Policy information about availability of computer code

| | |
|---|---|
| Data collection | AGO CLIP, ribosomal footprinting and RNA-seq samples upon AGO1 depletion have been generated for this study and are described in the method section. smallRNAseq samples, as well as RNA-seq samples from fly embryos and fly cell lines have been obtained from modENCODE, and are described in the method section |
| Data analysis | All data analyses are described in the method section. smallRNA-seq, RNA-seq and CLIP analysis data processing was done using public code and is described in detail in the method section. microRNA binding site prediction have been done using microMUMMIE. The code has been made available (see Code availability section) |

For manuscripts utilizing custom algorithms or software that are central to the research but not yet described in published literature, software must be made available to editors/reviewers. We strongly encourage code deposition in a community repository (e.g. GitHub). See the Nature Research guidelines for submitting code & software for further information.

## Data

Policy information about availability of data

All manuscripts must include a data availability statement. This statement should provide the following information, where applicable:

- Accession codes, unique identifiers, or web links for publicly available datasets
- A list of figures that have associated raw data
- A description of any restrictions on data availability

The AGO1 HITS- and PARCLIP data, small RNA-seq data, ribosomal footprinting data and RNA-seq data generated for this study have been submitted to the NCBI Gene Expression Omnibus (GEO; http://www.ncbi.nlm.nih.gov/geo/) under accession number GSE109980. Accession numbers and summary stats of previously published data sets analyzed in this study are present in Supplementary Data 16. Final miRNA binding site prediction are available in Supplementary Data files 8-13 and can be accessed https://dorina.mdc-berlin.de. The source data underlying Figs 1c and supplementary Figure 4B are provided as a Source Data file.

# Field-specific reporting

Please select the one below that is the best fit for your research. If you are not sure, read the appropriate sections before making your selection.

☒ Life sciences ☐ Behavioural & social sciences ☐ Ecological, evolutionary & environmental sciences

For a reference copy of the document with all sections, see nature.com/documents/nr-reporting-summary-flat.pdf

# Life sciences study design

All studies must disclose on these points even when the disclosure is negative.

| | |
|---|---|
| Sample size | See method section. 2 replicates AGO1 HITS-CLIP. 2 replicates AGO1 PAR-CLIP. 3 replicates ribosomal footprinting. 3 replicates RNA-seq. 4 replicates smallRNA-seq |
| Data exclusions | n/a |
| Replication | 2 replicates AGO1 HITS-CLIP. 2 replicates AGO1 PAR-CLIP. 3 replicates ribosomal footprinting. 3 replicates RNA-seq. 4 replicates smallRNA-seq. all samples are highly correlated. |
| Randomization | n/a |
| Blinding | n/a |

# Reporting for specific materials, systems and methods

We require information from authors about some types of materials, experimental systems and methods used in many studies. Here, indicate whether each material, system or method listed is relevant to your study. If you are not sure if a list item applies to your research, read the appropriate section before selecting a response.

## Materials & experimental systems

| n/a | Involved in the study | |
|---|---|---|
| ☐ | ☒ | Antibodies |
| ☐ | ☒ | Eukaryotic cell lines |
| ☒ | ☐ | Palaeontology |
| ☒ | ☐ | Animals and other organisms |
| ☒ | ☐ | Human research participants |
| ☒ | ☐ | Clinical data |

## Methods

| n/a | Involved in the study | |
|---|---|---|
| ☒ | ☐ | ChIP-seq |
| ☒ | ☐ | Flow cytometry |
| ☒ | ☐ | MRI-based neuroimaging |

## Antibodies

| | |
|---|---|
| Antibodies used | AGO1 abcam 5070 |
| Validation | used before e.g. in PMID: 23708604 |

## Eukaryotic cell lines

Policy information about cell lines

| | |
|---|---|
| Cell line source(s) | see supplemental methods |
| Authentication | n/a |
| Mycoplasma contamination | n/a |
| Commonly misidentified lines (See ICLAC register) | n/a |
